# Fresh, Mechanical, and Durability Behavior of Fly Ash-Based Self Compacted Geopolymer Concrete: Effect of Slag Content and Various Curing Conditions

**DOI:** 10.3390/polym14153209

**Published:** 2022-08-06

**Authors:** Aryan Far H. Sherwani, Khaleel H. Younis, Ralf W. Arndt

**Affiliations:** 1 Department of Civil Engineering, Faculty of Engineering, Soran University, Soran 44008, Kurdistan Region, Iraq; 2 Department of Civil Engineering, Fachhochschule Erfurt, University of Applied Sciences Erfurt, 99084 Erfurt, Germany; 3 Department of Surveying and Road Construction, Erbil Technology College, Erbil Polytechnic University, Erbil 44001, Kurdistan Region, Iraq; 4 Civil Engineering Department, Tishk International University, Erbil 44001, Kurdistan Region, Iraq

**Keywords:** self-compacting geopolymer concrete (SCGC), curing time and temperature, slag (GGBFS)/fly ash (FA), fresh properties, mechanical properties, modulus of elasticity, sorptivity, freezing and thawing

## Abstract

This investigation evaluates the influence of various curing conditions and slag inclusion on the fresh, mechanical, and durability properties of self-compacting geopolymer concrete (SCGC) based on fly ash (FA). Curing temperature and curing time have a vital role in the strength and microstructure of geopolymer concrete. Therefore, to begin the research, the impacts of different curing conditions (curing temperature and curing time) and slag content on the compressive strength of FA-based SCGC were examined to determine the optimum curing method. A series of four SCGC mixes with a fixed binder content (450 kg/m^3^) and an alkaline/binder ratio of 0.5 was designated to conduct a parametric study. FA was replaced with slag at four different substitution percentages, including 0%, 30%, 50%, and 100% of the total weight of the binder. The fresh properties of the produced SCGC specimens were investigated in terms of slump flow diameter, T50 flow time, and L-box height ratio. Additionally, the following mechanical properties of SCGC specimens were investigated: modulus of elasticity and fracture parameters. The water permeability and freezing–thawing resistance were studied to determine the durability behavior of SCGC. In this study, the optimum curing temperature was 85 °C for the duration of 24 h, which provided the maximum compressive strength. The results confirmed that adding slag affected the workability of SCGC mixtures. However, the mechanical characteristics, fracture parameters, and durability performance of SCGC were improved for slag-rich mixtures. When using 50% slag instead of FA, the percentage increase in compressive, flexural, elastic module, and fracture energy test values were about 100%, 43%, 58%, and 55%, respectively, whilst the percentage decrease in water permeability was 65% and the resistance to freeze–thaw test in terms of surface scaling was enhanced by 79%.

## 1. Introduction

Globally, cement use is increasing steadily, resulting in a considerable increase in carbon dioxide (CO_2_) emissions into the environment. Additionally, it uses a large number of raw materials for quarrying and minerals, which may lead to the prospective depletion of these materials in the future. Almost a ton of CO_2_ is released for every ton of ordinary Portland cement (OPC), depending on the manufacturing method used, which accounts for 5% of worldwide CO_2_ emissions [1,2]. The annual global production of OPC is estimated to contribute approximately 1.35 billion metric tons of carbon dioxide emissions [3,4]. By 2020, CO_2_ emissions from OPC manufacturing are predicted to be more than double the aforementioned levels [5]. To protect the atmosphere from the detrimental effects of cement manufacturing, it is critical to investigate renewable sources that can entirely or partially replace cement in concrete while causing no environmental degradation. To minimize the environmental footprint of cement production, a new binding material has recently been developed using a pozzolanic source binder that can be activated in an alkaline solution. This material is referred to as “geopolymer concrete” [6]. As a result, geopolymer concrete is viewed as the ideal option as it is not just a renewable source but it utilizes byproduct materials from the industry as a binder rather than natural raw material as OPC does [7]. In 1978, Davidovits postulated that binders may be synthesized by polymerizing alkaline liquids with silicon and aluminum in geologically derived or waste source materials such as fly ash (FA), ground granulated blast furnace slag (slag), or rice husk ash. He introduced the word geopolymer to refer to these binders [8]. The most commonly utilized industrialized by-product materials as binder ingredients are FA and slag [9,10,11,12,13,14,15]. 

Self-compacted concrete (SCC) is a superior category of concrete that may be consolidated completely into framework corners due to its own weight. SCC development intends to attain proper compaction and to assist the placing of concrete in congested elements and narrow openings [16,17,18,19]. SCC’s basic aspects include flowability, filling, and passage with no sign of segregation or bleeding [20]. Self-compacting geopolymer concrete (SCGC) is a novel phenomenon in concrete construction. The benefits of making SCGC are that it develops a concrete with combined geopolymer and self-consolidating features [21]. 

Alkali-activated or geopolymer concrete is an environmentally friendly material that also performs well in the hardened stage. Additionally, it consumes less energy during the manufacturing process and emits less CO_2_ than conventional concrete [22,23,24]. Geopolymer is an inorganic binder that has emerged as a viable alternative to OPC [25]. According to Srishaila et al. [26], the 56-day compressive strength of slag-based SCGC cured at ambient conditions is 40 MPa, but that of FA-based SCGC is only 16 MPa. Al-Rawi et al. [27] investigated the effect of slag on some fresh and mechanical properties of FA-based SCGC cured via oven. It was determined that adding slag to SCGC decreases its fresh properties but enhances its mechanical characteristics. The purpose of this experimental study is to investigate the influence of different slag contents on the fresh and hardened features of FA-based SCGC cured under different curing regimes.

The freeze–thaw effect is the most serious durability issue for concrete in cold climates. Dams and concrete pavement surfaces with large open zones are particularly vulnerable to frost in cold climates [28]. This deteriorating mechanism is not limited to cement-based concrete but also to geopolymer-based concrete. In cold weather, the water in cracks and capillary pores causes the degradation of geopolymer concrete. When the material is exposed to repeated cycles of freeze–thaw, the water in the cracks and capillary pores freezes. This incident causes internal pressure in the paste matrix. Internal microcracks are formed as a result of the expansion pressure. An increase in freeze–thaw cycles contributes to the extension of micro-cracks over time [29]. There are very limited investigations on the performance of geopolymer concrete and SCGC under freeze–thaw tests, and studies in this field need to be extended.

Curing conditions (or curing regimes) in terms of varying curing temperatures and curing times are the vital key parameters in the synthesizing of geopolymer concrete [30]. It has been noted that the curing regime has a substantial effect on determining the properties of geopolymer materials derived from by-products such as FA [31]. According to Palomo et al. [32], increasing the curing temperature led to an improvement in compressive strength. In contrast to OPC, the FA-based geopolymer concrete as a common type of binder displayed excellent mechanical and durability characteristics [33]. At elevated temperatures, geopolymer mixtures based on FA performed much better [34]. For sufficient geopolymerization of FA-based geopolymer concrete, a temperature range of 60–90 °C is necessary, which consumes a significant amount of energy. Concrete’s mechanical properties improve when it is properly cured [35]. The maximum compressive strength was attained for the FA-based SCGC after 48 h of a curing regime at 70 °C. A 48 h curing time is considered optimal, as no substantial contribution to compressive strength was detected after 48 h [36]. Calcium oxide (CaO) additionally generates hydration compounds, including calcium-silicate-hydrates (C-S-H) and alumino-silicate geopolymer gel, which lead to an enhancement in the strength of geopolymer concrete [37,38]. Slag incorporation into geopolymer concrete based on FA caused accelerated setting times and increased strength, resulting in a concrete that could be cured in ambient conditions [39]. At room temperatures, geopolymeric materials based on FA react relatively slowly and typically exhibit a slow rate of setting and strength development [40]. Therefore, due to the high flowability of SCGC, the setting time and strength gain in the early age of FA-based SCGC could be a critical consideration. Incorporating slag may be a viable option for resolving this issue. However, according to earlier studies, there is still a shortage of studies on the role of curing condition on the strength of SCGC-based on FA with varying slag concentrations. 

Table 1 highlights a review of the literature on the experimental work of plain SCGC manufactured with or without FA and slag as well as the current investigation. From the literature reported above and listed in Table 1, there are a few or a lack of studies investigating the influence of slag content on some mechanical and durability characteristics of SCGC. Moreover, based on the curing conditions (curing time and temperature), the majority of the research emphasizes a limited curing regime of FA-based SCGC, whereas the impact of the combined influence of slag content and various curing conditions on the strength of FA-based SCGC has not been studied yet. For the reasons stated above, the objective of this study can be separated into two parts. The impact of curing conditions on the strength of SCGC specimens made with four replacement levels of FA with slag by the total volume of binder (0, 30, 50, 100%) was investigated in the first step. After the mixing and casting operations were completed, the specimens of SCGC were subjected to different curing conditions and then tested for compressive strength. For this step, the curing conditions were room curing, step curing, and oven curing at 40 °C for the duration, which ranged between 3 to 28 days. To some extent, some of the specimens were subjected to an elevated temperature of 60 °C for the duration of 24 and 48 h, and oven curing at 85 °C was also used on SCGC specimens for periods of 4, 8, 12, 14, 24, and 48 h. A delay time was also included as a parameter (1 hour and 24 h) at an elevated temperature of 85 °C. Then, based on the maximum strength test values of the SCGC specimens examined in the previous step, oven curing at 85 °C for 24 h was chosen as the best curing regime to explore the influence of slag inclusion on the various fresh and hardened attributes of FA-based SCGC specimens. Slump flow diameter, T50 flow time, and L-box height ratio were the fresh tests conducted in this work. Additionally, the mechanical features of SCGC as expressed by its compressive strength, modulus of elasticity, and fracture parameters were determined. Different durability properties of the produced geopolymer concrete specimens, including water permeability and freezing–thawing resistance, were also studied. 

## 2. Materials Utilized

To produce self-compacting geopolymer concrete (SCGC), class F fly ash (FA) was utilized as a binder according to DIN EN 450-1 [61]. In the production of SCGC, ground granulated blast furnace slag (slag) was also used as a binder in this investigation, which was provided by the Dyckerhoff GmbH company. Table 2 shows the physical characteristics and chemical composition of FA and slag as provided by the supplier.

MC Power flow evo 502 is a polycarboxylate ether-based superplasticizer (SP) that meets the requirements of EN 934-2: T3.1/3.2 which was used. It has a yellowish color and a density of 1.03 kg/m^3^. For the activation process in geopolymer concrete, a variety of alkaline solutions are commonly utilized. The alkaline solution (AL) utilized in this experiment was a mixture of sodium-silicate (Na_2_SiO_3_) and a sodium-hydroxide (NaOH) solution. The mass of sodium silicate (Na_2_SiO_3_) used in the production of SCGC was 45% dry content and 55% water content. Table 3 shows the chemical and physical features of Na_2_SiO_3_. The sodium hydroxide (NaOH) employed in this study has a purity of 99%. By dissolving the NaOH solid particles in water, the NaOH solution was created. The molarity (M) of NaOH is used to express its concentration. The molarity used in this study was fixed at 12 M. 

The water was utilized in this study to improve workability. The water utilized in the experiments was clean tap water that was readily available in the civil engineering lab at FH Erfurt. 

The natural coarse aggregates provided by the Amagard company were river gravel. The gravel size ranged from 16.0 to 4.0 mm, and the fine aggregates used in the SCGC mixes were river sand. The physical characteristics of gravel and sand were tabulated in Table 4.

## 3. Concrete Mixing Procedure 

Four mixes of self-compacting geopolymer concrete (SCGC) were designated with an alkaline/binder (AL/B) ratio of 0.5 and a total binder content of 450 kg/m^3^ [41]. Water was fixed at 40 kg/m^3^ for the entire experimental study. These mixtures were designed with different slag contents with the same aggregate content, binder content, molarity (M), superplasticizer (SP), and water. Slag was used as the only parameter in this stage to replace the FA at different percentage levels (0%, 30%, 50%, and 100%). Furthermore, the effects of slag on various properties of the FA-based SCGC were investigated. Table 5 illustrates the detailed mixed design proportions in kg/m^3^ of the SCGC made in this study. In the following mix codes, G0 indicates that the binder content is 100% FA and 0% slag. 

### 3.1. Mixing, Casting, and Sample Preparation

A similar mixing procedure was used to achieve consistent homogeneity and uniformity in each mixture. In the first stage, all the dry ingredients, such as coarse and fine aggregates, FA, and slag as a binder, were mixed in the electrical concrete mixer, with a 75-litre capacity and a rotation speed of 70 rpm, for about 2 min. After mixing the dry ingredients, the premixed liquids of AL and water (mixed for one hour prior to mixing time) were introduced to the mixer and continued for 2 more minutes. Then, the superplasticizer was supplied to the wet batch and the mixing procedure proceeded for an additional 2 min. The temperature of the SCGC mixtures ranged between 26 to 28 °C. The SCGC fresh batch was tested for flowability, viscosity, and passing ability properties after the mixing process was completed, and the specimens for hardened state properties were then prepared. The fresh concrete was mixed again in the mixer for half a minute before the casting stage to maintain the homogeneity of the mix. The specimen shapes were cubical specimens (100 × 100 × 100 mm, 150 × 150 × 150 mm, and 150 × 150 × 75 mm), cylindrical specimens (Ø150 × 300 mm), and beam-like specimens (100 × 100 × 500 mm), and they were cast with no means of compaction due to the self-consolidating property under their own weight. Moreover, after casting, the specimens’ surfaces were scraped using a finishing trowel to eliminate excessive material and achieve a smooth surface. Finally, the cast specimens of SCGC were placed under ambient curing for the curing process to begin.

### 3.2. Curing Condition

**Figure 1:**
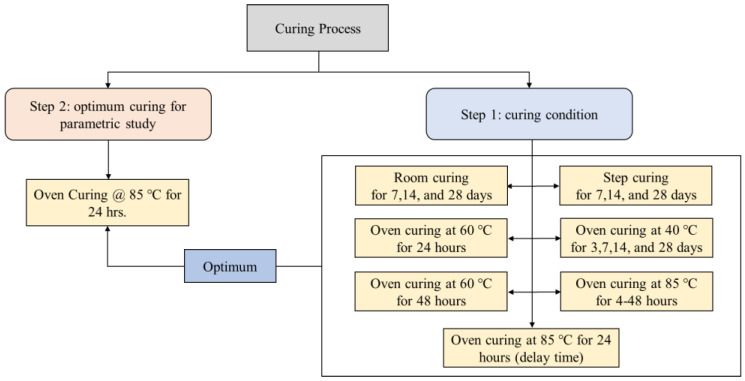
Diagram for the curing conditions and selecting optimum curing of SCGC mixtures.

The curing process of SCGC specimens starts immediately after the mixing and casting stages are completed. Before being exposed to curing, the samples were stored in the lab for 24 h [64]. In this study, in the first step, different curing methods were examined on the SCGC specimens, and subsequently, the cured specimens were assessed for compressive strength (see Figure 1). The optimal curing type was then chosen based on the compressive strength values to measure the different properties examined in this research study. For each test measurement, an average of three cubes of 100 mm in size were taken using the compression machine of 3000 kN capacity referred to as BS EN 12390 [65]. The following are the classifications of curing conditions implemented for the mixed groups:

#### 3.2.1. Room Curing 

SCGC specimens were kept at room temperature (22 °C with a relative humidity of 60 ± 5%) until the testing date. If the requirement for minimum compressive strength for structural applications can be achieved, this method would be the most preferable one related to low energy consumption. SCGC specimens in room curing were kept for the duration of 7, 14, and 28 days before testing for strength.

#### 3.2.2. Step Curing 

A digitalized UIM 800 oven machine manufactured by the MEMMERT GmbH company was used to apply step curing to specimens. The step curing cycle lasts 24 h per day; 9 h at room temperature, 2 h of rising heating temperature to reach the target degree of 40 °C and maintain that degree for 11 h, and finally, end the cycle with 2 h of cooling down in the chamber. This curing method continued for 7, 14, and 28 days (cycles), and the samples were taken out of the oven for two hours before testing.

#### 3.2.3. Oven Curing 

For the oven curing or heat curing method, three curing temperatures, such as 40, 60, and 85 °C, were selected. A digitalized oven is used for curing SCGC specimens at a constant temperature of 40 °C for 3, 7, 14, and 28 days. Moreover, the specimens are kept at ambient condition for 2 h before testing. Furthermore, an electric oven set to a maximum temperature of 300 °C was used to apply 60 °C to the SCGC samples for the periods of 24 and 48 h, then kept at an ambient temperature until the testing date (7 days of age). Another method of elevated heat curing is to place the specimens in an electric oven set to 85 °C for a period of 24 h. The delay time was also examined in this approach. The delay time refers to the time period before the specimens are placed in the oven [66]. A 24 h delay time was included in all curing conditions. However, in the case of the 85 °C curing temperature, the effect of a one-hour delay period was also investigated during this phase. After heat curing, the samples were stored at room temperature until the testing date (7 days). Furthermore, the SCGC samples were also placed under 85 °C for the curing times of 4, 8, 12, 14, 24, and 48 h after one day of casting, and then, prior to testing specimens for compressive strength, the specimens were stored at ambient temperature for two hours before being subjected to compressive force.

Figure 1 demonstrates that 85 °C for 24 h is the optimum curing regime which provides the maximum compressive strength (as will be justified in Section 5.1.6), and is applied to the parametric study including mechanical and durability properties conducted in this study. After the curing procedure was completed, the samples were stored at ambient conditions for 28 days.

Percentage increases in compressive strength can be calculated based on the following formula:

(1) $$Percent\;increase\;in\;f_{cu}\;at\;Gi=\frac{f_{cu}\;at\;Gi-f_{cu}\;at\;G0}{\;f_{cu}\;at\;G0}\times 100$$

where *f_cu_ at Gi* is compressive strength at various slag content (i.e., 30%, 50%, or 100%), and *f_cu_ at G*0 is compressive strength with no slag (100% FA). 

## 4. Testing Procedure

### 4.1. Fresh Properties 

Fresh tests were performed on the mixtures of SCGC conducted in this investigation after the mixing process to meet EFNARC’s guidelines [67]. The flowability of SCGC mixtures was tested using slump flow diameter tests. The T50 flow time at which the flow diameter of the fresh concrete reaches 50 cm is also measured. The passing ability of the freshly mixed SCGC is determined by means of the L-box test, which is the ratio of the height of the concrete in the horizontal section to the height of the concrete in the vertical section after the flow has stopped.

### 4.2. Mechanical Properties 

**Figure 2:**
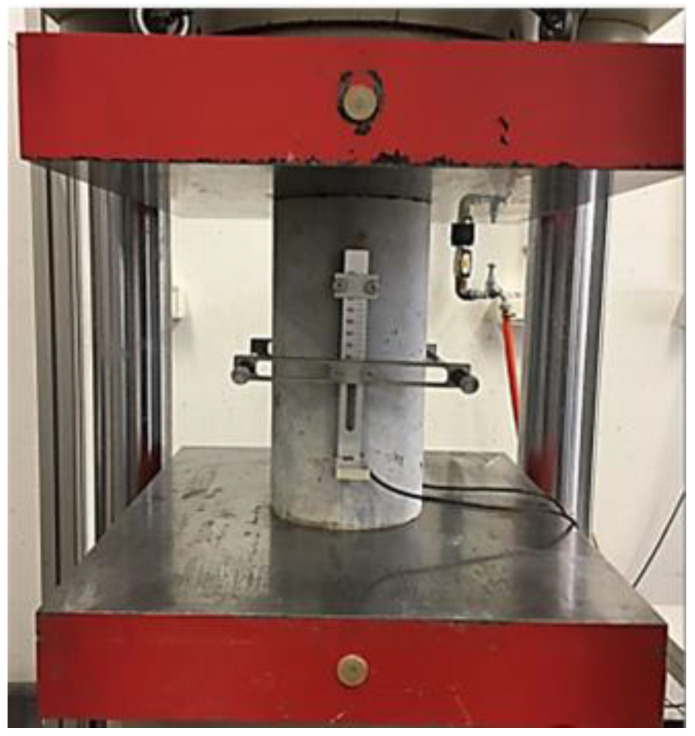
Modulus of elasticity test: strain gauge set up.

An average of three cylinders (Ø150 × 300 mm) were taken at 28 days of age to determine the static elastic modulus of elasticity (Es), confirming EN 12390-13 [68]. The casting side of the cylindrical specimens was grinded and polished by a concrete specimen grinding machine before being subjected to the elastic modulus test. Two strain gauges were used to perform the test, and the test sample was positioned in the center of the testing machine with the measuring instruments attached axially, as can be seen in Figure 2. In total, three loading cycles were applied, and the first and second cycles were discarded, while the static elastic module was determined by the third cycle.

### 4.3. Fracture Parameters 

**Figure 3:**
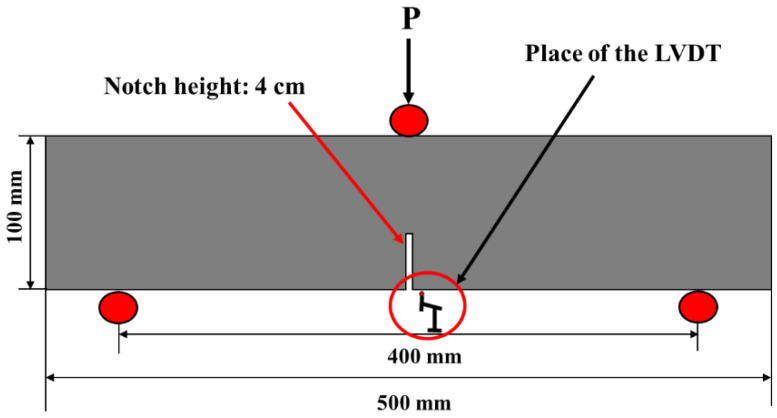
Principal diagram of bending test.

To implement the flexural strength test, an average of three 100 × 100 × 500 mm beams were taken for each measurement, confirming the RILEM 50-FMC/198 committee [69]. During this test, the fracture parameters for SCGC specimens were computed. Simultaneous displacement measurements were carried out at the beam’s midspan using an LVDT (linear variable displacement transducer) placed at the beam’s center point. A universal flexural testing equipment with a 250 kN capacity with a loading rate fixed at 0.02 mm/min was used. Figure 3 illustrates the schematic testing set up in detail. Moreover, beam specimens were evaluated at 28 days of age. Before testing day, the beam was notched in the center to measure the displacement at the center of the beam. Moreover, the notch was created by reducing the effective cross section to 60 × 100 mm using a cutter machine. Thus, the proportion of the notch height to beam depth (a/W) was 0.4. However, the span of the beam was fixed at 400 mm. Equation (2) was utilized to determine the net flexural strength, whereas P_max_ is defined as the highest load that the beam can support without regard for notch sensitivity [70,71].

(2) $$\mathrm{Net}\;\mathrm{flexural}\;\mathrm{strength}\;=\frac{3P_{\max}S}{2B{\left(W-a\right)}^{2}\;}$$

After obtaining the load-displacement curve at the midspan of the beam specimens, the fracture energy (*G_F_*) was estimated using the following formula:

(3) $$G_{F}=\frac{W0+\mathrm{mgA}}{\mathrm{BL}}$$

where A = $\delta_{s}\;\frac{s}{u}\;$; and L = (W−a) represents the region underneath the load-deflection curve (N.m), m is the beam weight (kg), g is the gravitational acceleration (9.81 m/s^2^), δ is the specified displacement of the beam (m), S is the span length (mm), U is the length of the beam (mm), B is the width of the beam (mm), W is the depth of the beam (mm), and a is the notch depth of the beam (mm). 

### 4.4. Durability Properties 

#### 4.4.1. Water Permeability

SCGC specimens were tested for water permeability using the BS EN 12390-8 standard [72] to determine the water penetration depth for the hardened SCGC specimens after 28 days. For this purpose, three cubical specimens of 150 × 150 × 150 mm in dimensions were tested for each measurement. The water pressure was applied to the bottom side of the specimens at 500 ± 50 KPa for the duration of 72 h. When the time periods were finished, the cubic specimens were removed from the device, the dry face exposed to the applied water pressure, and then split in the middle section to measure the maximum water penetration depth. 

#### 4.4.2. Freezing and Thawing 

**Figure 4:**
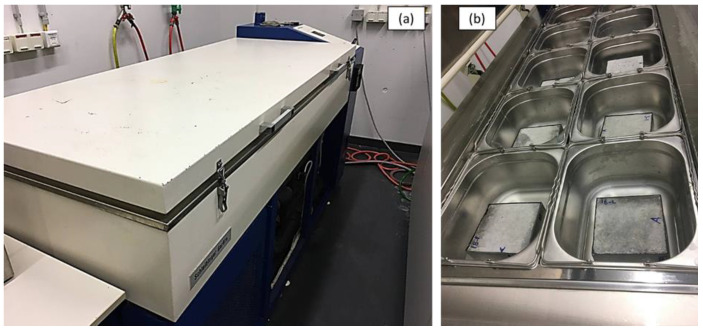
Freezing–thawing test device: (**a**) freezing–thawing chamber, (**b**) specimens test set up.

Freezing and thawing tests were applied on SCGC specimens according to TC 117-FDC [73]. The test procedure comprises three steps: storing specimens in dry condition, pre-saturating specimens by capillary force, and starting cycles of freeze–thaw. The test requires four specimens with dimensions of 150 × 150 × 75 mm [74]. G0 and G50 were chosen as the typical mix categories. After curing, the specimens’ lateral sides must be wrapped with a latex membrane to prevent the sides from absorbing water. The specimens are then positioned in test tubes with spacers of 5 mm in height and a 150 × 150 mm test surface. Following that, water is put into the container with an average height of not more than 10 mm and the lid is closed within the duration of capillary suction. This procedure requires seven days of suction at a temperature of 22 °C. Later, the testing technique is subjected to repeated cycles in a chest container. The bath temperature was regulated by the temperature control system. To achieve this, an automatic testing machine (Schlesinger CIF freezing and thawing test machine) is utilized to perform the proper thermal cycles (see Figure 4). The parameters are determined at temperatures greater than 15 °C. Throughout 14 days, the machine performs a freeze–thaw cycle (28 cycles). Using an ultrasonic water bath, the scaled material on the side of the concrete samples that is exposed to freeze–thaw cycles is removed. 

Measurements of SCGC samples are performed at the start of the freeze–thaw test and after each 6th freeze–thaw cycle. The testing procedure should determine scaling, water absorption, and interior damage. To gather the scaled material, the filter paper was utilized.

After filtering the scaled material in the solution, the mass of the scaled material (*μ_b_*) in the dry state is recorded with 0.0001 g accuracy. Before filtering, the mass of the filter (*μ_f_*) is measured. The scaled material (*μ_s_*) in grams is then measured as follows:

(4) $$\mu_{S}=\mu_{b}-\mu_{f}$$

After the nth cycle, Equation (5) was used to calculate the overall scaled material removed from the test side region for the selected specimens.

(5) $$m_{n}=\frac{\sum \mu_{s}}{A}\times 10^{6}$$

where *m_n_*, *μ_s_*, and *A* are the total mass of collected scaled material following each measurement period, in relation to the surface area of tested specimen (g/m^2^), the quantity of scaled material per each measurement (g), and the area of the test surface area (mm^2^). Following the nth cycle, the following equation is utilized to compute the relative increase in mass of each sample (Δ*w_n_*):

(6) $$\Delta w_{n}=\frac{w_{n}-w_{1}+\sum \mu_{s}}{w_{0}}\times 100$$

where Δ*w_n_*, *w*_0_, *w*_1_, and *w_n_* denote the weight of moisture absorption (uptake) of each sample specimen following the nth cycle in percentage, the reference weight of each sample (g), each reference sample with the weight of sealed material (g), and the weight of each sample for each selected cycle (g). 

Internal damage occurs when the microstructure of concrete deteriorates, resulting in a change in the characteristics of the concrete. The RILEM TC 176 method [73] was used to detect the interior damage of concrete samples. The relative dynamic modulus of elasticity (RDME) was determined using ultrasonic transit times based on relative transit time. The RDME in percentage after 28 cycles of freeze and thaw can be found by the following equation:

(7) $$\mathrm{RDME}\;\left(\%\right)={\left(\frac{t_{cs}-t_{c}}{t_{n}-t_{c}}\right)}^{2}\times 100$$

where *t_cs_* is the total transit time at the end of capillary suction (*cs*) in ms before starting the test, *t_n_* is the total transit time after 28 cycles of freeze–thaw, in ms, and *t_c_* is the transit time in the coupling medium in ms. 

## 5. Results and Discussions 

### 5.1. Curing Condition 

Based on the curing conditions, geopolymer concrete can display various types of properties and behaviors. For synthesized geopolymers, the curing phase is a critical parameter. In this section, the influence of curing conditions, which comprise curing temperature and curing time, on the compressive strength of fly ash (FA)-based self-compacted geopolymer concrete (SCGC), was investigated. The effect of replacing FA with ground granulated blast furnace slag (slag) was also investigated. For this reason, four mixes with various slag content (0%, 30%, 50%, and 100%) were selected. The cube samples (100 × 100 × 100 mm) were subjected to different curing conditions, including curing at room temperature, step temperature, 40, 60, and 85 °C for a variety of curing times, and then tested for their compressive strength.

#### 5.1.1. Room Curing

**Figure 5:**
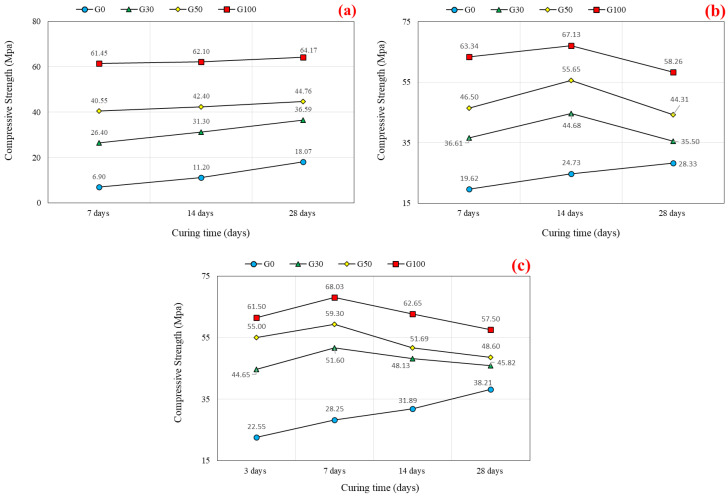
Compressive strength versus curing time: (**a**) room curing, (**b**) step curing, and (**c**) oven curing at 40 °C.

**Figure 6:**
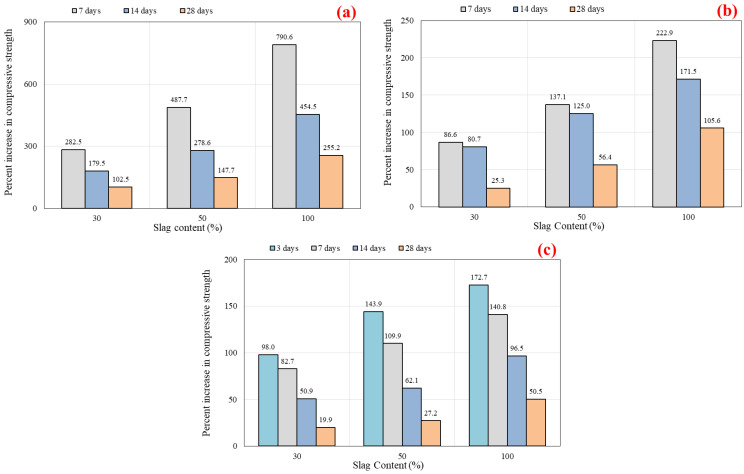
Percentage increase in compressive strength versus slag content: (**a**) room curing, (**b**) step curing, and (**c**) oven curing at 40 °C.

Figure 5a illustrates the compressive strength values (MPa) of SCGC versus curing time (days) for the four replacement levels of FA with slag (0, 30, 50, and 100%). Moreover, Figure 6a presents the compressive strength increment rate with respect to slag content. The SCGC specimens were cured at room temperature for a 7-, 14-, and 28-day curing periods. It was discovered that as the content of slag increases, so does the compressive strength, irrespective of the curing days. At 7 days of age, the lowest value for the compressive strength of SCGC was 6.90 MPa for 100% FA content, whereas the highest value was attained (61.49 MPa) for the mix made with 100% slag. For geopolymer concrete specimens containing FA that are cured at room temperature, the chemical reaction occurs slowly, resulting in a slow hardening process and slow strength development. Therefore, higher temperatures are expected to activate the alumino-silicate elements in the FA. They are typically cured at elevated temperatures (ranging from 60 °C to 90 °C) [40]. This conclusion is corroborated by the findings of Kirschner et al. [75], who asserted that ambient curing was infeasible due to setting delay.

At 7 days of age, based on the reference mix (G0), the rate of strength increment with increasing slag content is higher than that of 14 and 28 days. For instance, with the increment of slag addition at the following ratios of 30%, 50%, and 100% of the total binder content, the compressive strength increased at the following rates: 282.5%, 487.7%, and 790.6%, respectively, with regard to the 7 days of age (See Figure 6a). Whereas the increment rate was decreased to 179.5%, 278.6%, and 454.5% for the 14 days of age and 102.5%, 147.7%, and 255.2% for the 28 days. Due to the lesser calcium levels in the FA binder compared to the slag, the G0R0 mix has low strength gain, and the G30, G50, and G100 mixes have high strength gain at 7 days. Previous research has also revealed similar behavior [76]. At ambient conditions, the combination of FA and slag as a precursor in the synthesis of geopolymer concrete that CaO was involved with led to an increase in strength development. This was achieved by both hydration and geopolymerization [77]. Overall, the outcomes indicate that the inclusion of slag in geopolymer concrete mixes results in enhanced initial setting and strength gain, whereas the use of FA leads to a decrease in initial setting and strength [39]. Li et al. [78] revealed that the development strength of slag-based geopolymer concrete is quicker than FA-based geopolymer material. This might be due to the existing calcium compounds in the composition of slag that promote initial setting and the slow leaching of FA particles at ambient curing [9,79,80]. It was declared that the presence of CaO in slag encourages the hydration process, so a 100% slag content mixture has more compressive strength [79]. Ismail and Bernal [80] observed that calcium-rich pastes typically form a C-S-H gel, which forms a dense microstructure and increases strength. Nevertheless, as Si increases and calcium decreases, it forms an N-A-S-H gel or creates a composite C-N-A-S-H gel, resulting in a loss of strength. The higher the slag percentage in the FA and slag-based mixture, the greater the dissolved alumino-silicates. While curing at ambient temperatures, Nath and Sarker [9] found that slag generates internal heat in the mix that aids the geopolymerization process and leads to considerable strength gains. Therefore, when geopolymer concrete mixes are cured at room temperature, the strength growth follows the same pattern as that of ordinary Portland cement (OPC) concrete. This is a novel advancement in terms of on-site usage and the elimination of heat curing in geopolymer concrete. However, the optimal content of slag employed was not mentioned, and no other hardened properties were investigated at this curing condition. 

**Figure 7:**
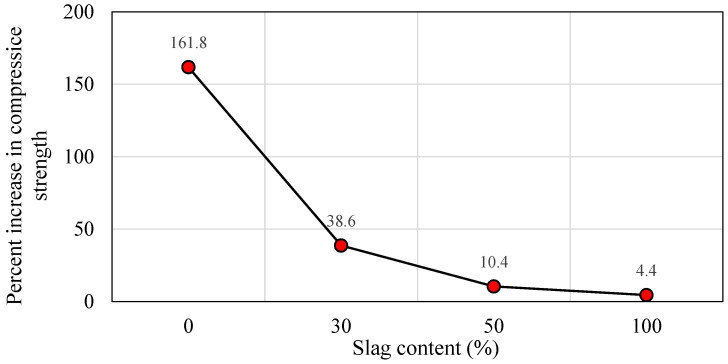
Percentage increase in compressive strength test results vs. slag content for 28 days compared to 7 days curing.

Besides, a prolonged curing time provides a lower improvement in the compressive strength than a shorter curing time. This was observed for the SCGC mixes that are rich in slag compared to the mixes made with 100% FA (G0R0). Figure 7 illustrates the compressive strength development rate in percentage for 28 days curing compared to 7 days curing. This is linked to the substantial early-age strength growth usually achieved when slag is utilized. It may be stated that when the slag content grows, the effect of concrete strength gain with age decreases. For example, the G0 had a 161.8% increase in compressive strength at 28 days, while the G30, G50, and G100 had 38.6%, 10.4%, and 4.4% strength increments, respectively, when compared to the results at 7 days.

#### 5.1.2. Step Curing

The compressive strength behavior of SCGC for different curing times is shown in Figure 5b. The effects of slag content on the FA-based SCGC were investigated for three curing times (7, 14, and 28 days). Step curing is performed by a digitalized oven at the desired controlled temperatures. Temperature variations on the specimens were implemented in the oven range of 22–40 °C, which corresponded to nighttime and daytime outdoor temperatures. This method was implemented in order to implement and simulate air curing rather than expose specimens to day and night temperature variations in the open air. With this curing method, the maximum compressive strength of 67.13 MPa was attained for the G100 at 14 days. The compressive strength values of the SCGC mixes with slag contents of 0%, 30%, 50%, and 100% were 19.62 MPa, 36.61 MPa, 46.50 MPa, and 63.34 MPa, respectively, for the 7 days of curing. A slight increment in strength is recorded up to 14 days of curing, followed by a strength declination beyond that limit. However, the mix of 100% FA content had a different trend of strength development as it was increasing proportionally with its curing time. This could be explained by the fact that there is insufficient moisture in the slag content mixes to allow for further strength development due to the rapid initial setting and strength gain. In a different study, it has been demonstrated that geopolymer concrete specimens require an adequate amount of moisture for heat curing [66]. Moreover, it was reported that extending heat curing time for the geopolymer mixture promotes strength development during the first 24 h of hardening. For instance, after curing at 40 °C for an hour, the strength of a geopolymer mortar was found to be only 13 MPa. When the curing period was prolonged to four hours, the strength nearly tripled to 37 MPa. However, there is no significant strength development at 7 days of age with regard to elevated curing [81]. Furthermore, it was declared in a previous study that for a 90-day curing time, the mix made with 60% of slag mix showed the highest strength instead of the mix made with 100% slag content, compared to the outcomes recorded at 28 days. As the hydration reaction proceeds in the slag for a prolonged period of time, the specimen dehydrates continuously, resulting in crack formation. As a result, up to a specific slag content in the FA and slag mix, improved strength is noted, but above that, a decrease in strength is recorded [79]. 

The rate of strength growth increased as the slag content increased, irrespective of the curing period, as shown in Figure 6b. However, the biggest percentage increase occurred at 7 days curing, followed by 14 and 28 curing days. For instance, based on 7 days of age, with adding the slag content at the ratios of 30%, 50%, and 100% of total binder content, the rate of increment in compressive strength results was 86.6%, 137.1%, and 222.9%, respectively. Furthermore, as the curing time was increased (14 and 28 days), the rate of strength development decreased.

#### 5.1.3. Oven Curing at 40 °C

Geopolymer concrete curing at room temperature has a compressive strength that is insufficient in comparison to concrete mixes curing in an oven. This is due to the alkaline solution and binder starting to partially polymerize at an initial age. Additionally, heat curing was required to create a rapid geopolymerization technique capable of achieving an appropriate strength in a very short time, which is a critical criterion for the synthesis of geopolymers [10,11]. The cube specimens were cured in an oven at 40 °C for the duration of 3, 7, 14, and 28 days and were tested to evaluate the effects of various slag contents (0%, 30%, 50%, and 100%) on the compressive strength of FA-based SCGC (See Figure 5c). This curing method was implemented to see the behavior of SCGC at low curing temperatures. From the results, it was concluded that the SCGC values improved as the slag content increased. At 3 days of age, the compressive strength values were 22.55 MPa, 44.65 MPa, 55.00 MPa, and 61.50 MPa for the slag content ratios of 0%, 30%, 50%, and 100%, respectively. With a prolonged curing time to 7 days of oven curing, the compressive strength reached its maximum limit (28.25 MPa, 51.60 MPa, 59.30 MPa, and 68.03 MPa). Then, the compressive strength began to decline as the curing time was prolonged for the following 14 and 28 days, except for geopolymer concrete mixes containing 100% fly ash which exhibited the reverse behavior. Therefore, 7 days curing at 40 °C seems to be preferable to 14 and 28 days. It is widely established that FA-based geopolymer concrete requires heat curing to achieve adequate early mechanical strength qualities, which might be a significant constraint for on-site applications [82]. Moreover, it was concluded that the extended curing period improved the geopolymerization mechanism and resulted in increased strength [83]. According to Rovnanik [81], the elevated temperature has a substantial effect on the hardening and geopolymerization of rock-based geopolymers. On a rock-based geopolymer exposed to heat curing between 40 and 80 °C, an increase in strength development was observed. It is essential to remember that the influence of temperature is dependent on the curing time. Curing for a shorter period of time in the oven had no significant effect on strength development, but extending the curing progression to a minimum of 20 h resulted in a substantial increase in reaction rate and early strength acquisition. 

Figure 6c demonstrates the percentage increment rate of compressive strength for the various curing times. G0 is defined as a reference line in the study for the percent increase rate. When utilizing 30%, 50%, and 100% slag content, the increases in strength development rates were 98.0%, 143.9%, and 172.7%, respectively, for 3 days curing time. The percentage increment rate is enhanced with the utilization of slag. The highest increment rate was referred to the early age (3 days) and then decreased with extending curing time for 7, 14, and 28 days of age. From the findings, the strength development rate mostly occurred at early ages for the mixes that were rich in slag, irrespective of the curing time. Moreover, the strength enhancement decreased as the curing duration exceeded 7 days for the mixes including slag, whereas for the FA-SCGC mixes, the strength increment rate increased as the curing period extended. Likewise, slag-rich mixes exhibit greater strength values than the FA-based mixes after a specific curing period. There is no such considerable increase in strength with increasing curing duration for mixes containing higher slag content than FA. In addition, fly ash reacts at a slower rate than slag [79]. Existing calcium oxide in the chemical composition of slag enhances the rapid setting, resulting in early strength [9,78]. 

#### 5.1.4. Oven Curing at 60 °C

**Figure 8:**
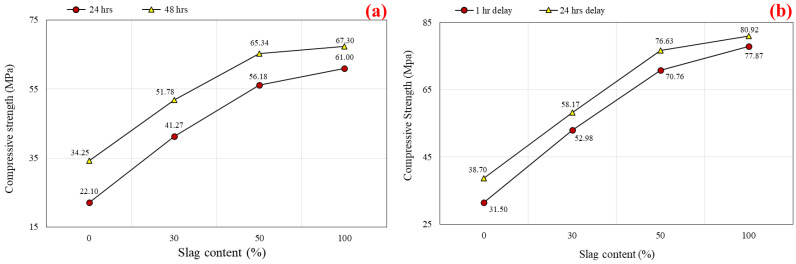
Effect of slag content on the compressive strength of SCGC specimens: (**a**) oven curing at 60 °C for 24 and 48 h; and (**b**) oven curing at 85 °C for 24 h with 1 and 24 h delay times.

**Figure 9:**
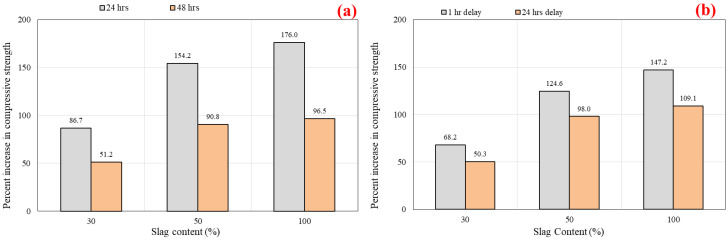
Percentage increase in compressive strength vs. slag content: (**a**) oven curing at 60 °C for 24 and 48 h; and (**b**) oven curing at 85 °C for 24 h with 1 and 24 h delay times.

**Figure 10:**
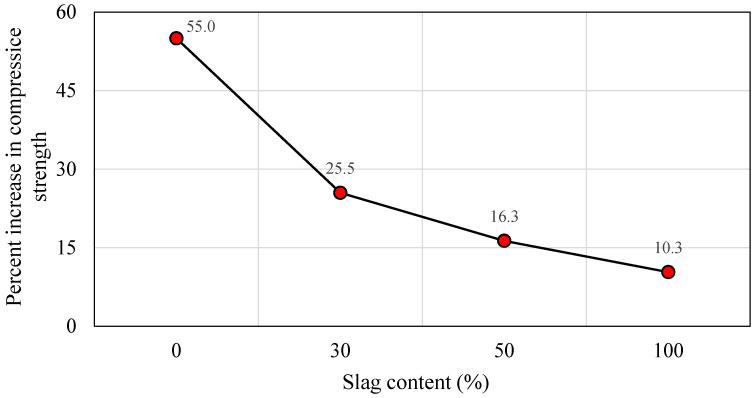
Percentage increase in compressive strength versus slag content for 48 h compared to 24 h curing time.

Geopolymer materials require heat treatment to achieve a compressive strength equivalent to or superior to that of normal concrete. This form of curing is advantageous for the dissolving and repolymerizing of the silica and alumina gel and also leads to an increase in the material’s early-age strength [84,85,86,87]. In this section, the results of the specimens subjected to oven curing at 60 °C are presented. Figure 8a displays the impact of various slag inclusions on the compressive strength of SCGC for different curing hours. Furthermore, the samples were oven cured for 24 and 48 h separately, and then they were tested at 7 days of age. Regardless of the curing time, a large improvement in compressive strength was observed up to 50% slag addition, accompanied by a steady increase as the slag addition reaches 100%. At a 24 h heat curing process, the compressive strength values were 22.10 MPa, 41.27 MPa, 56.18 MPa, and 61.00 MPa for the slag content of 0%, 30%, 50%, and 100%, respectively. Additionally, it was noticed that the compressive strength grew as the slag level in the geopolymer mix enlarged. The highest compressive strength was obtained for a geopolymer concrete made entirely of slag without the addition of fly ash (44 MPa) [82]. Figure 9a explains the increment rate in compressive strength versus different slag content for 24 and 48 h curing times. From Figure 9a, the development rate was calculated based on the reference mix (100% FA). A remarkable growth in strength was detected as the proportion of slag grew. The growth of compressive strength for 24 and 48 h heat curing ranged between 86.7–176.0% and 51.2–96.5%, respectively. It is observable that the development rate in compressive strength is very high up to 50% CR content, and beyond that limit, there is no remarkable growth in strength, regardless of the curing time. In general, the rate of geopolymerization increases with increasing slag and activator concentrations in slag mixed binders [9,88]. Moreover, for the duration of 48 h, the highest strength development rate was recorded (55%) for 0% slag content compared to 24 h of oven curing, while the increment in strength was decreased to 10.3% as the slag level reached 100%, as shown in Figure 10. Therefore, it is obvious from the test findings that as the slag level increases, the strength development rate decreases with prolonged curing time. The above variations refer to the fact that the slag-based mixes were developing higher strength levels at initial curing ages compared to the FA-based mixes. This pattern was previously observed in the curing experiments conducted in this investigation and is described above. 

#### 5.1.5. Oven Curing at 85 °C

In terms of early strength development, the elevated heat curing process has been shown to be the best approach for curing geopolymer concrete. This form of curing, however, is challenging to achieve in cast-in-place applications [89]. However, the samples typically lose moisture content at elevated curing temperatures, but geopolymerization requires moisture to attain a high compressive strength. Thus, previous research indicates that curing temperatures greater than 90 °C results in a decrease in the compressive load of geopolymer concrete [66]. Therefore, the highest temperature that was implemented in this investigation is 85 °C. The impact of delay time on the strength was also proposed as shown in Figure 8b. For this purpose, 1 h and 24 h were selected as the delay times in this test. From the 7-day test result, the increase in strength was found to be directly related to the amount of slag in the mixture. When extending the delay time from 1 h to 24 h, the strength of the SCGC specimens was improved, regardless of the slag content. However, the highest compressive strength was 80.92 MPa for 100% slag content when the delay time was 24 h. Lim et al. [66] reported that compressive strength without delay time is found to be approximately 31.5 MPa. The maximum strength of 33 MPa was achieved after the delay time was raised to one hour. This is attributed to the reason that the delay time permits the alumina and silica to dissolve as the primary elements for alumino-silicate geopolymerization. However, when the delay duration exceeds 24 h, a significant change in strength occurs. In addition, a slight delay in the beginning of oven curing could increase the strength of geopolymer concrete. This could be because the process of geopolymerization is finished before heat curing begins [66]. Based on the above findings, a 24 h delay time was implemented in this study before being subjected to the curing condition. 

**Figure 11:**
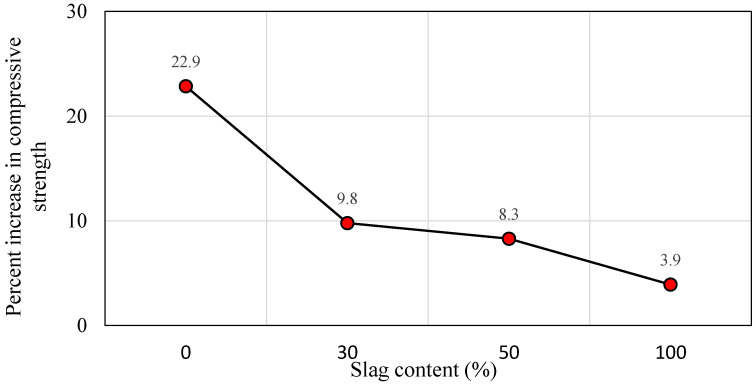
Percentage increase in compressive strength test values vs. slag content for 24 h of delay time compared to 1 h.

Likewise, the strength increment rate for the 1 h and 24 h delay times with respect to the slag content is displayed in Figure 9b. The compressive strength is significantly enhanced with the increase in the slag content. For instance, for the 1 h delay time, the compressive strength increment rate was 68.2%, 124.65, and 147.2% for the slag ratios of 30%, 50%, and 100%, respectively. Then, for the comparable mixes, a diminishing trend in strength development was detected as follows: 50.3%, 98.0%, and 109.1% as the delay time was prolonged to 24 h. To some extent, the rate of increase in compressive strength was maintained at a high level until the slag content reached 50% replacement level. As the slag level surpassed that threshold, a little increase in strength was noted. Additionally, the percentage variations in compressive strength for the 24 h delay time compared to the 1 h delay time versus slag content is depicted in Figure 11. The mixes that are rich in FA exhibited better improvement in percent increase in compressive strength than the mixes that are rich in slag. The maximum and minimum rates of increase in compressive strength were 22.9% and 3.9%, for the slag contents of 0% and 100%, respectively. Besides, the slag rich mix exhibits greater strength values than the fly ash mixes after a short curing period. There is no such considerable increase in strength with increasing curing time for mixes containing higher slag [79]. Furthermore, it is clear from Figure 11 that delay time is more effective for the FA-based geopolymer concrete specimens than the slag-rich mixes. 

**Figure 12:**
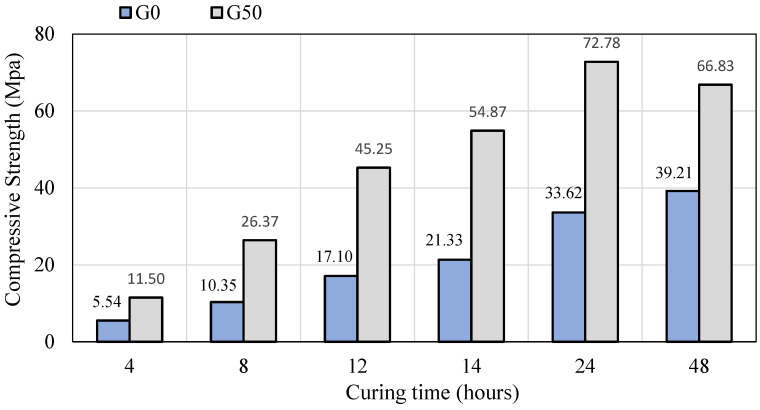
The effect of curing time at 85 °C on the compressive strength of SCGC mixes with 0% and 50% slag content.

The compressive strength of SCGC specimens was also studied in relation to the duration of heat curing at 85 °C, as illustrated in Figure 12. The G0 and G50 mixes of SCGC were cured for various curing times (4, 8, 12, 14, 24, and 48 h), followed by cooling the specimens by storing them at room temperature for 2 h before compressive testing was performed. For the FA-based SCGC mix, improvement in the compressive strength readings ranged from 5.54 MPa to 39.21 MPa when the curing period was prolonged. Similarly, for the SCGC produced with 50% slag, the compressive strength was enhanced by increasing the curing time up to 24 h of heat curing, and the maximum value was 72.78 MPa. Beyond that limit, the compressive strength results begin to drop, so that heat curing of G50 mix at 48 h declined to 66.83 MPa. This trend might be due to the non-existence of enough moisture for strength development. Liquid is released during the geopolymerization reaction of geopolymer concrete and is prone to evaporating when specimens are heated during curing [48]. It was also stated that after 24 h of curing at 70 °C, a significant change in strength was noted. Compressive strength decreased as a result of prolonged curing times [40]. For the same reason, it has been argued that prolonged curing of geopolymer concrete will only weaken its microstructure, resulting in a fall in compressive strength [90,91]. As a result, in actual applications, the length of heat curing should not exceed 24 h [66]. Therefore, in this study, curing for the duration of 24 h at 85 °C seems to be preferable for the FA and slag blended SCGC. This type of curing can be very efficient for pre-cast applications to achieve high strength at elevated temperatures for a short period of time. 

#### 5.1.6. Optimum Curing Condition

**Figure 13:**
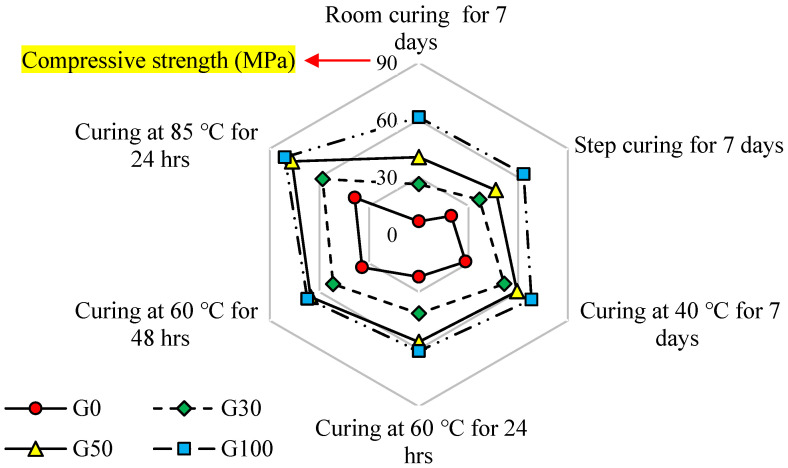
Radar chart of variations in compressive strength (at 7 days in MPa) vs. various slag content and curing methods.

Figure 13 depicts the variations in 7 days compressive strength (MPa) of SCGC-based on FA for various curing techniques and slag content. In the aforementioned figure, the specimens cured via room, step, oven at 40 °C, oven at 60 °C, and oven at 85 °C were conducted and tested at 7 days of age. At overall evaluation, a lower compressive strength was found in SCGC samples that had been cured at low temperatures than in those that had been cured at elevated temperatures. The influence of curing time and temperature was greater for the strength development of the FA-based SCGC mixes than on the slag-based SCGC mixes. On the contrary, the slag-rich mixes yield greater strength values in the study, regardless of the curing time and curing temperature. According to the test results shown in Figure 13, the optimum curing method used throughout the study is oven curing at 85 °C for 24 h, which provides the highest compressive strength. Furthermore, the purpose of this study is to utilize a higher rate of FA with a combination of slag that provides good mechanical and durability behavior. Regarding the type of binder used, the mix made with 100% FA obtained the smallest compressive strength, whilst the mix made of 100% slag recorded the highest strength value. However, as explained in Section 5.1.1, Section 5.1.4 and Section 5.1.5, the strength growth for the samples having 100% slag content compared to the mix made with 50% slag content is very small. In light of these findings, it is safe to say that 50% FA and 50% slag are the ideal binder ratios (G50) for SCGC mixes.

### 5.2. Fresh Properties 

The influence of incorporating slag on the performance of fly ash (FA)-based self-compacted geopolymer concrete (SCGC) mixes was examined and the results were compared with the limitations of EFNARC recommendations [67]. The fresh properties of group mixes were measured by assessing flowability, viscosity, and passing ability through the slump flow diameter, T50 flow time, and L-box height ratio, respectively. The outcomes of the fresh behavior of the SCGC mixes with various slag additions are illustrated in Table 6. According to the results, it appears that using more slag in the SCGC mixes causes a systematic decrease in the slump and L-box test results of SCGC. However, the T50 flow time values increased with the increase in slag content. SCGC mixtures with higher slag contents resulted in lower flowability and passing ability, as well as higher viscosity. Therefore, SCGC mixtures with higher slag content were found to be stickier (more viscous) and more cohesive. Furthermore, slag has been claimed to be more reactive than FA due to its physical and chemical properties [92]. As a result, it was utilized as a blended binder with FA in this study to determine its impact on the SCGC in a fresh and hardened state. Slag increased the water consumption of concrete when compared to FA particles due to its finer particle size and higher specific surface area. Consequently, the flowability and passing ability of concrete in a fresh state is diminished with an increase in viscosity. Apart from the above findings, all the fresh values of SCGC presented in Table 6 were within the EFNARC limitations [67]. 

### 5.3. Mechanical Properties 

#### 5.3.1. Static Modulus of Elasticity (Es)

**Figure 14:**
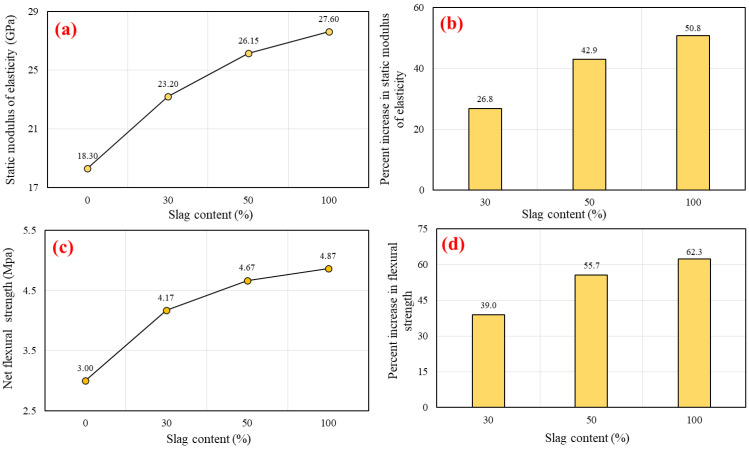
Influence of slag replacement level on the: (**a**) static modulus of elasticity, (**b**) percent increase in static modulus of elasticity, (**c**) net flexural strength, and (**d**) percent increase in net flexural strength.

The Young modulus is a critical property of a material that is used in the structural concrete design parts because it provides vital information about the ability of concrete to resist deformation at its elastic limit [93,94]. The 28-day test values of the static modulus of elasticity (E_s_) versus various slag levels are depicted in Figure 14a. The E_s_ values in this investigation ranged from 18.3 to 27.6 GPa. The SCGC mixes with the use of slag had a considerably greater E_s_ compared to the mixes without slag. The peak E_s_ result was attained for the geopolymer concrete mix produced with 100% slag content (G100R0). The result values demonstrate that the E_s_ were increased by using more slag. The same behavior was also observed in the compressive strength test. Li et al. [95] established that the E_s_ is highly reliant on the physical parameters of the concrete and the utilized aggregate (such as density and porosity). Nath and Sarkar [96] investigated the mechanical properties of geopolymer concrete based on FA. In their research, it was discovered that E_s_ was enhanced as the concrete strength increased. It was detected that the E_s_ of geopolymer concrete is comparatively lower than that of normal concrete of similar strength. Normal concrete had an E_s_ of 30.6 GPa at 28 days, corresponding to 40 MPa of compressive strength. Additionally, for the parallel strength of geopolymer concrete, the E_s_ obtained values ranged between 21.6 and 23.2 GPa for the same testing age. This is nearly 30% lower than the result of PC concrete. Furthermore, the elastic modulus of geopolymer concrete was found to be much lower than that of normal concrete, even though the compressive strength of geopolymer concrete is comparable to or higher than that of normal concrete. As a result, it is possible that geopolymer binders can sustain more deformations in an elastic state than OPC binders [97]. Figure 14b illustrates the percent increase in static modulus of elasticity of SCGC with various slag contents compared to the reference mix (100% FA). It was detected that E_s_ values increase by 50.8% when the slag content is increased from 0% to 100%, whereas the highest development rate in the static modulus of 43% was achieved as the slag addition reached 50% of total binder content. Hager et al. [98] also found that the specimens of geopolymer concrete based on FA mixed with slag had a higher elastic modulus than the mix made with only FA. It has also been reported that the E_s_ of geopolymer concrete improves with increasing compressive strength, but it is significantly less than that of conventional concrete with equivalent compressive strength. [99,100]. 

#### 5.3.2. Flexural Strength

The net flexural strength of the SCGC mixes was measured by Equation (2) and shown in Figure 14c. Indeed, the flexural strength of SCGC was greatly improved by increasing the slag replacement level instead of FA. Increment in the flexural strength might be due to improving the bond between the aggregate and the geopolymer paste. The net flexural strength values obtained were 3.00 MPa, 4.17 MPa, 4.67 MPa, and 4.87 MPa for the mixes with various slag content (0%, 30%, 50%, and 100%, respectively). This study’s findings are in line with those of prior investigations into the flexural strength of SCGC produced with FA and slag [27,41]. The percent increase in flexural tensile strength of the SCGC mixtures is presented in Figure 14d. The flexural strength development rate was 39.0%, 55.7%, and 62.3% for the slag contents of 30%, 50%, and 100%, respectively, compared to the control mix (100% FA). 

### 5.4. Fracture Parameters 

#### 5.4.1. Load vs. Displacement 

**Figure 15:**
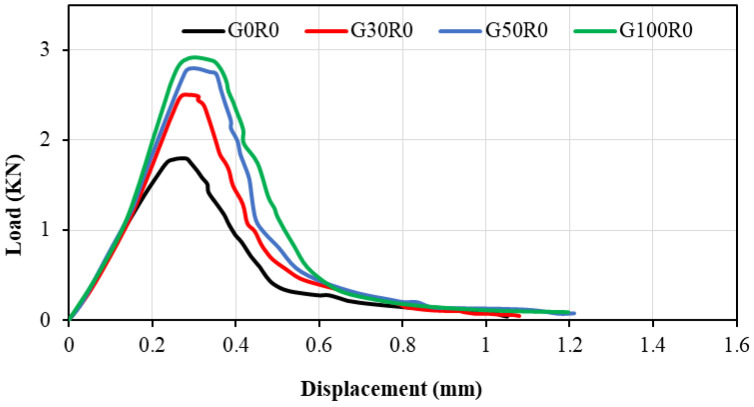
SCGC load vs. displacement charts relative to slag content at 28 days.

The SCGC load vs. displacement profile for the prisms with various slag replacement levels (0%, 30%, 50%, and 100%) is shown in Figure 15. SCGC curves generally have a linear ascending slope until the stress reaches the first crack in specimens. During the test procedure, cracks form when the load exceeds the peak load, which is the maximum load on the load–displacement curve, resulting in a declining curve following the peak load. On the other hand, the slope of the descending half of the curve after peak load showed the crack propagation characteristics inside the specimen until failure. In Figure 15, the maximum and minimum peak loads are 1.8 and 2.92 kN for the mixes having slag content of 0% and 100%, respectively. Additionally, the outcomes indicate that the peak load values and load vs. displacement of SCGC specimens were enhanced with an increasing replacement level of FA with slag. This might be due to the fact that mixes with slag exhibit higher strength when compared to the control mix (100% FA). A similar pattern was described for the SCGC made with blended FA and slag in a previous study [27]. Moreover, the peak load achieved in the study ranged between 1.4 to 2.72 kN for the 100% FA and 100% slag content, correspondingly [27].

#### 5.4.2. Fracture Energy (*G_F_*)

**Figure 16:**
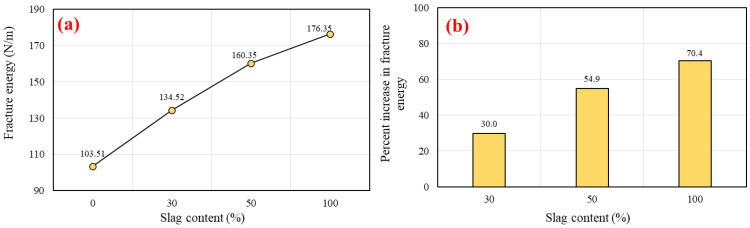
Impact of slag replacement level on the: (**a**) fracture energy, and (**b**) percentage increase in fracture energy.

Despite the importance of fracture energy (*G_F_*) in determining the ultimate stress at the crack tip, it is a function of displacement rather than strain. It is defined as the amount of energy necessary to open a fracture surface of a unit area [101]. Additionally, the fracture characteristics of concrete define its ductility behavior. The higher value of *G_F_* indicates the concrete’s greater ductility [102,103]. The fracture energy was calculated by Equation (3). In this study, the *G_F_* outcomes of SCGC versus various slag content are illustrated in Figure 16a. The values revealed that the utilization of slag binder in the SCGC mixes significantly enhanced the *G_F_* compared to the reference mix made with 100% FA. For instance, the mix with 100% FA recorded 103.51 N/m as the minimum value of *G_F_* conducted in this investigation, whereas the maximum value of *G_F_* of 176.35 N/m was obtained for the mix made with 100% slag. This behavior for the FA and slag blended SCGC was also reported by other studies [27]. The percentage increase in fracture energy (see Figure 16b) was 30.0%, 54.9%, and 70.4% for the mixes with replacement levels of FA with slag of 30%, 50%, and 100%, respectively, with respect to the control mix (G0R0). The *G_F_* increment rate of the mixes beyond 50% of slag content seems to be a steady increase, whereas a sharp increase in *G_F_* was seen for the replacement level of FA with slag of up to 50%. These characteristics were also noted in the mechanical properties investigated in this investigation. Generally, as SCGC compressive strength increases, the fracture energy tends to improve. Midhun et al. [104] and Sarker et al. [105] demonstrated that the *G_F_* of FA-based geopolymer concrete cured via oven improved with increasing compressive strength. 

### 5.5. Durability Properties 

#### 5.5.1. Water Permeability 

**Figure 17:**
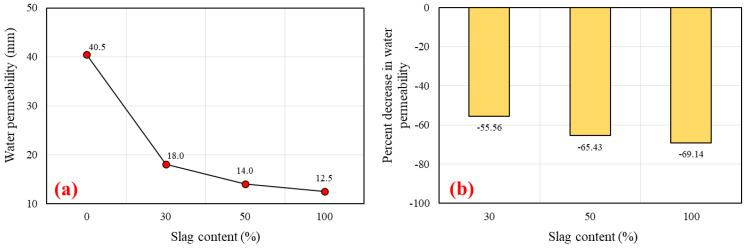
Impact of slag replacement level on the: (**a**) water permeability of SCGC, and (**b**) percentage decrease in water permeability.

**Figure 18:**
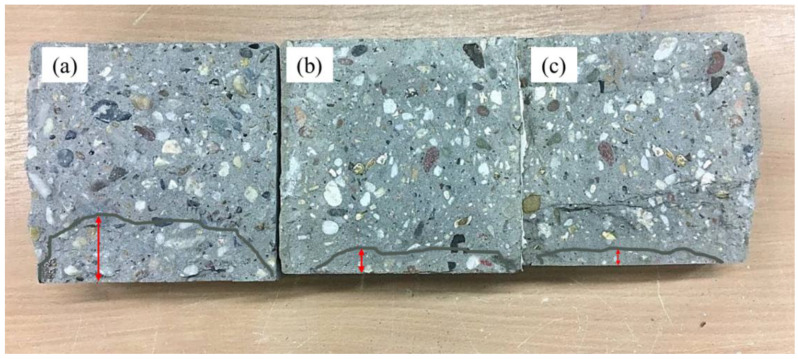
A cross-sectional view of depth of penetration of water for the split specimens after 72 h subjected to water pressure; (**a**) 100% FA, (**b**) 30% slag, and (**c**) 50% slag.

Water permeability measures the depth of water that can flow due to pressure and capillary absorption. This test measures the water permeability rate and its effect on the degree of geopolymerization, which are highly important parameters. The water permeability in terms of depth of water penetration (mm) is shown in Figure 17a. The rate of permeability decreased as the slag content increased. The minimum and maximum values of depth of penetration of 40.5 mm and 12.5 mm occurred for the 100% FA and 100% slag content mixes, respectively. In a previous study, it was revealed that the penetration depth for geopolymer concrete was lower than for normal concrete. The depth of penetration for the geopolymer concrete ranged between 31.2 mm and 35.7 mm [106]. It was also found that the water permeability of geopolymer concrete is not primarily determined by porosity but also by the alkaline solution proportion [106]. The percent decrease in water permeability was 55.56%, 65.43%, and 69.14% in comparison to the control mix (G0R0) as the replacement level of FA with slag increased from 0% to 30%, 50%, and 100%, respectively (See Figure 17b). A higher degree of geopolymerization results in a smaller void fraction and a SCGC matrix that is less permeable to water. Water permeability appears to decrease with age in geopolymer concrete due to the continued geopolymerization process [107]. The sorptivity of slag-rich mixtures is lower than that of mixtures containing 100% FA. In addition, an increase in compressive strength due to the increase in slag content confirmed it. The permeability of a material depends on the pore structure, size distribution, and continuity of pores. This new gel formation changed the pore structure and densified the microstructure by filling these pores. As the age increased from 1 day to 28 days, the pores were gradually occluded by the production of geopolymer gel and C-S-H gel. Incorporating slag into SCGC promotes the production of N-A-SH gel [45]. A photographic view of the tested specimens being split to measure the depth of water penetration is presented in Figure 18.

#### 5.5.2. Freezing and Thawing

##### Surface Scaling 

**Figure 19:**
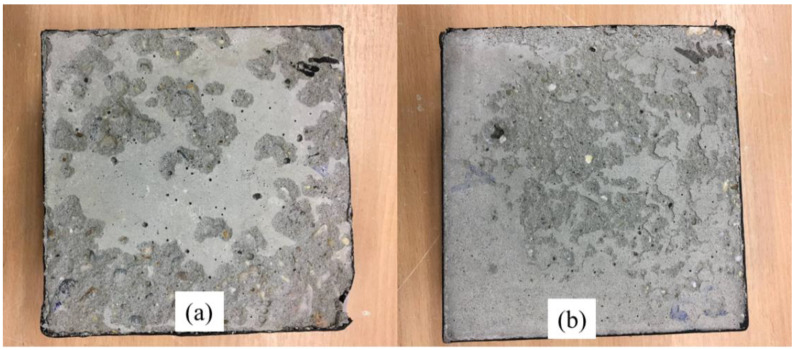
Photographic view of tested specimens after 28 freeze–thaw cycles; (**a**) 0% slag content, (**b**) 50% slag content.

The results of the SCGC samples exposed to 28 cycles of freeze–thaw using distilled water are discussed in this section. According to test values, the minimum values of the surface scaling were achieved with the utilization of 50% slag. This might be related to the higher compressive strength of the slag and FA blended SCGC specimens in comparison to the reference mix. The result values for the 0% slag and 50% slag were 325 and 68 g/m^2^, respectively. The percentage decrease in results was 79% as the slag content increased from 0% to 50%. Previously, it was stated that the higher compressive strength of concrete led to less surface scaling. The allowable range for surface scaling is 1500 gr/cm^2^ following the RILEM TC117-FDC [74]. For instance, the surface scaling for high and normal strength concrete exposed to freeze–thaw and deicing salt ranged approximately between 65 and 350 g/m^2^, individually [108]. It is generally established that concrete comprises a variety of voids. Freeze and thaw damage occurs when water inside the capillary pores of concrete freezes. The photographic view of specimens subjected to 28 cycles of freezing and thawing is presented in Figure 19. Moreover, Pilehvar et al. [109] studied FA and slag-based geopolymer concrete in comparison to normal concrete. They concluded that geopolymer concrete displays superior properties against freezing and thawing cycles than normal concrete.

##### Moistures Uptake

The results of moisture uptake were also conducted in this study. The test values displayed a comparable trend to the scaling outcomes. Clearly, the increase in the pore system led to an increase in the SCGC samples’ moisture absorption rates. This impact can be linked to the insufficient ability of FA-based SCGC compared to the slag rich mixes. The moisture uptake values achieved ranged between 1.45 and 3.58 in mass percentage. For high strength concrete, a moisture uptake of less than 0.5 was achieved, whereas higher values were recorded for normal concrete [108]. 

##### Internal Damage

As described in the CIF testing procedures, the damage condition is lower than 80% [73]. The relative dynamic modulus of elasticity (RDME) values after 28 cycles of freeze–thaw testing are explained below. All concrete types exceed the damage threshold. However, the 50% slag content specimen yields the greatest RDME value. The RDME results were 95.5% and 99% for the mixes having 0% and 50% slag content, respectively. This is an indication that the slag content specimens display superior properties to the FA-based specimens. Karakurt and Bayazit [108] recommended that to increase the resistance of concrete against freeze–thaw, the addition of air entraining to concrete could be a suitable option.

## 6. Conclusions

In this investigation, the effects of different curing conditions (curing temperature and time) and slag content were studied on the compressive strength of FA-based self-compacted geopolymer concrete (SCGC) to determine the optimum mixture and curing regime. Furthermore, the mechanical and durability properties of SCGC mixes were investigated. The following are the major conclusions:

1. In all curing conditions (including room curing, step curing, oven curing at 40 °C, oven curing at 60 °C, and oven curing at 85 °C), replacing FA with slag positively affects the strength of the SCGC. When the slag content increases, the compressive strength of the SCGC also increases, regardless of the curing temperature. Prolonged curing time has a remarkable influence on the strength development of FA-based SCGC mixes compared to mixes having slag content. One-day delay time is essential for SCGC specimens prior to being subjected to oven curing.
2. Optimum curing condition: based on the compressive strength, 24 h of oven curing at 85 °C has been found to be the optimum curing condition.
3. Optimum combination binder: in geopolymer SCGC mixtures, the majority of strength growth occurred at 50% slag inclusion. Beyond that content, strength improvement is insignificant.
4. The slump flow diameter of SCGC mixes was greatly affected by the replacement level of FA with slag. Adding slag causes a dramatic reduction in slump flow diameter. However, all the results were within EFNARC’s limits.
5. A systematic increase in the static modulus of elasticity of SCGC specimens was detected as slag content increased. With the increasing slag level from 0% to 100%, the elastic module values increased by about 50%, from 18.30 to 27.60 GPa.
6. The net flexural strength values enhanced as the percentage of slag level increased compared to the reference mix (100% FA). The net flexural strength values were 3.00 MPa and 4.87 MPa for the slag replacement levels of 0% and 100%, respectively.
7. The fracture energy of SCGC specimens is enhanced with the increase in slag inclusion. As slag content increased from 0% to 30%, 50%, and 100%, the percent increase in fracture energy was 30%, 54.9%, and 70.4%, respectively.
8. The water permeability of SCGC was enhanced as the slag content increased. Increasing slag content from 0% to 100% causes a considerable reduction in water pentation depth from 40.5 mm to 12.5 mm.
9. Freeze–thaw resistance of SCGC specimens improves with the rise in compressive strength and also increases with the increase in slag content. As slag inclusion increases from 0% to 50%, the surface scaling reduces from 325 g/cm^2^ to 68 g/cm^2^, respectively.

## Figures and Tables

**Table 1:** The SCGC in the current study is compared to those in these previous studies.

Refs	Composite Type	Binder (kg/m^3^)	Binder Type	Curing Regime	Mechanical Properties	Durability Properties
[27]	SCGC	450	FA (100, 75, 50, 25, 0%) slag (0, 25, 50, 75, 100%)	70 °C for 48 h	Compressive, splitting, net flexural, Load/displacement, fracture parameters	-
[41]	SCGC	450	FA (50%), slag (50%), NS ^1^ (5–10 kg/m^3^)	70 °C for 48 h	Compressive, bond strength, flexural, fracture parameters	-
[42]	SCGC/ SCAAC	480	FA (25%), slag (75%)	Ambient	Compressive	Chloride penetration, water penetration, capillary, Abrasion, Acid-sulphate attack, shrinkage
[43]	SCGC	450, 500	slag, NS (9–10 kg/m^3^)	60 °C for 24 h	Compressive, flexural, bond strength	Sorptivity
[44]	SCGC	475	FA (Class F & C)	Ambient	Compressive	Sulphate-acid attack, capillary, chloride penetration, corrosion
[45]	SCGC	500	FA (100%), slag (100, 95, 85, 75%), RHA ^2^ (5, 15, 25%)	Ambient	Compressive, splitting, flexural	Sorptivity
[46]	SCGC	500	FA (100%), slag (100, 95, 85, 75%), RHA (5, 15, 25%)	60 and 70 °C for 24 h, Ambient	Compressive, splitting, flexural	-
[47]	SCGC	436	FA	60 °C for 24 h	Compressive, splitting	-
[48]	SCGC	400	FA	60, 70, 80, and 90 °C for 24, 48, 72, and 96 h	Compressive	-
[36]	SCGC	400	FA	60, 70, 80, and 90 °C for 24, 48, 72, and 96 h	Compressive	-
[6]	SCGC	400	FA	70 °C for 48 h	Compressive	-
[49]	SCGC	424	FA (100, 80, 60, 40, 20, 0%), slag (100, 80, 60, 40, 20, 0%)	70 °C, Ambient	Compressive, splitting, flexural strength	-
[21]	SCGC	450	FA	60, 75, 85, 90 °C for 24 and 48 h	Compressive	-
[50]	SCGC	400	FA (100, 95, 90, 85, 80%) MK (5, 10, 15, 20%) GSA ^3^ (5, 10, 15, 20%)	75 °C for 48 h	Compressive, splitting, flexural strength	Water permeability
[51]	SCGC	396	MK ^4^	Closed plastic bag	Flexural	-
[52]	SCGC	475	slag	Ambient	Compressive, splitting, flexural	Carbonation depth, drying shrinkage, acid resistance,
[53]	SCGC	400	FA	70 °C for 48 h	Compressive, splitting, flexural	-
[54]	SCGC	450	FA (50%), slag (50%)	Ambient	Compressive	-
[55]	SCGC	450	FA (100–70%), slag (10, 20, 30%), SF ^5^ (5, 10, 15%)	70 °C for 48 h	Compressive, splitting, flexural	-
[56]	SCAAC	500	Slag (100, 98%), NS (2%)	Ambient	Compressive, splitting, net flexural, Load/displacement, modulus of elasticity	-
[57]	SCGC	400	FA	70 °C for 24, 48 h	Compressive	-
[58]	SCGC	400	FA (100, 90, 80, 70%), MK (10, 20, 30%)	70 °C for 24 hours	Compressive, splitting, flexural strength	-
[59]	SCGC	400	FA (100, 90%), SF (10%)	70 °C for 48 h	-	Drying shrinkage
[60]	SCGC	484	Slag (100, 70, 60, 50, 40, 30%) FA (30, 40, 50, 60, 70%)	27 ± 1.5 °C (75% relative humidity)	Compressive, splitting, flexural strength	-
Current study	SCGC	450	FA (100, 70, 50, 0%) slag (0, 30, 50, 100%)	Step 1: Various curing time and temperature. Step 2: 85 °C for 24 h	Compressive, net flexural strength, modulus of elasticity, load/displacement, fracture energy	Water permeability, freeze–thaw

Where ^1^ NS is nano silica, ^2^ RHA is rice husk ash, ^3^ GSA is groundnut shell ash, ^4^ MK is metakaoline, ^5^ SF is silica fume.

**Table 2:** Chemical composition and physical properties of FA and slag.

Component %	CaO	SiO_2_	Al_2_O_3_	Fe_2_O_3_	MgO	SO_3_	K_2_O	Na_2_O	Various	Specific Gravity	Loss on Ignition	Blain Fineness (cm^2^/g)
FA	4	55	23	7.0	2.0	-----	2.0	1.0	6.0	2.22	3.0	3098
Slag	40.06	36.24	11.0	0.44	7.63	1.28	0.83	0.30	2.22	2.80	2.30	4250

**Table 3:** Chemical and physical features of Na_2_SiO_3_.

Na_2_O (%)	SiO_2_ (%)	Water Content by Mass (%)	Viscosity (mPas) (20 °C)	Density (g/cm^3^) (20 °C)	pH
15.0	30.0	55	550	1.55	12.5

**Table 4:** Physical characteristics of gravel and sand.

Type of FA	Size (mm)	Specific Gravity	Water Absorption (%)
Gravel	4.0–16.0	2.58	0.52
Sand	0.0–4.0	2.54	0.81
Standard	BS EN 933-1 + A1 2005 [62]	BS EN 1097-6:2013 [63]	BS EN 1097-6:2013 [63]

**Table 5:** Mix design of SCGC.

Mix Code	Binder (kg/m^3^)	FA (%)	Slag (%)	Gravel (kg/m^3^)	Sand (kg/m^3^)	AL/Binder	Molarity (M)	SP (%)	Water (kg/m^3^)
G0	450	100	0	800	825	0.5	12	7	40
G30		70	30	800	825	0.5	12	7	40
G50		50	50	800	825	0.5	12	7	40
G100		0	100	800	825	0.5	12	7	40

**Table 6:** Fresh test results of the SCGC.

Mix Code	Slag (%)	Slump (cm)	T50 (sec)	L-Box Height Ratio
G0	0	78.8	2.1	1.00
G30	30	77.5	2.3	0.99
G50	50	75.0	2.9	0.96
G100	100	68.0	4.1	0.84
